# Mixed Training

**Published:** 1907-05-11

**Authors:** 


### Nursing Administration.

### MIXED TRAINING.

The system under which nurses have for many years been trained at the Hospital for Consumption, Brompton, constitutes a very interesting experiment in co-operation between general and special hospitals. The hospital, by its size and also by the national character of the work carried on within its walls, affords in certain directions unique opportunities for instructing probationers. With the continual growth of sanatoria for consumptives there arises an increasing demand for nurses accustomed to the distinctive treatment required by the phthisical patient. Further, for the spread of the common-sense tenets relating to personal hygiene, on which the life of the patient in the early stages of the disease may be said to depend, the doctor must rely on nurses thoroughly grounded in the principles and practice of the best modern treatment for tuberculosis. The hospital at Brompton has 319 beds, 108 being at Frimley, and a nursing staff of 74, so that, deducting the nursing staff engaged mainly outside the wards, there is an average of 4.2 beds to each member of the day and night staff. Salaries are given to probationers on the following scale:—First year, £10; second year, £12; third year, £24; rising £2 per annum to £30. During the second year of service, however, the probationer, though receiving a salary from the institution, passes out of its service to a general hospital.

On the completion of this year of service and instruction in a general hospital, the nurse returns to complete her engagement at Brompton, and usually, though bound for a period of only three years, remains an extra year from a sense of the benefits received, and with a view to fit herself more thoroughly for her future work. How does this mixed training tell in the development of a good nurse, and in what way is it found possible to arrange the probationer's instruction, so that part of it may be given in the special institution, and part at a general hospital? The latter question is, perhaps, the most difficult to answer. For the former it may be said that the dislocation of routine implied in the abrupt change in the probationer's outlook at the end of her first year is not found to work harmfully in any particular. There is, perhaps, less difference between the medical wards in different hospitals, than between the accident ward and obstetrical ward in one and the same hospital. The probationer is accustomed, from the very outset of her hospital career, to startling changes of scene and to no less complete changes of method and discipline between one ward sister and another. It is more than likely that she may early awake to the fact that what is a fixed law in one ward, and under certain doctors, is merely a pious opinion held with some degree of scepticism by others. Hence there is nothing particularly disquieting to her when the time comes for her to pass, not merely from one floor to another, but from a special hospital to an institution of a wider character, in the course of her training. From the point of view of disposition it is, in many instances, all for the good. Every matron knows the probationer who is apt to grow a little "stale," after the first stretch of hospital life has been gone through. A thorough change of scene and discipline may often have a very bracing effect, in cases such as this, nor has a keen nurse ever been known to deteriorate under a new regime. As regards both theoretical and practical work, it can be readily recognised that a special hospital under able administration offers opportunities for covering a very large field. The theoretical instruction of the probationer commonly comprises lectures on anatomy, physiology, hygiene, surgical nursing and medical nursing. The first three subjects can obviously be taught equally well in a special hospital, and a wide range of medical nursing is afforded by a hospital which deals mainly with diseases of the circulatory and respiratory systems. All the general duties of the medical nurse can be learned in such wards, including the management of the ward, the care of the patient, the observation of the patient, the administration of medicines, the use of fomentations, poultices, packs, enemata, catheters, etc., the care of bedding, the preparation of baths, the use of the thermometer, and, in fact, all the processes with which the private nurse must be familiar in the sick room. Even in the domain more strictly appertaining to surgical work there is much to be learned in such an institution as the Brompton Hospital. The principles of asepsis, and the properties of antiseptics; the treatment of congestion, bedsores, hæmorrhage; the preparation of bandages, the cleaning and sterilisation of instruments—these are only a few of the subjects, usually taught under the heading of surgical nursing, which could be well studied by the nurse in many special hospitals. Gaps in the knowledge of nurses thus trained there must inevitably be, unless the training is supplemented in a general hospital. But when it is calculated that a third of the time spent in hospital is under this scheme given to surgical nursing, and to the medical work not already included in the course, it does not appear that the training comes far short of that received in the ordinary general hospital without any break. For if the time which the probationer spends in the various departments be split up, it will be often found that she spends little more than a year, after all, in wards where acute surgical and medical cases are received. The making of the nurse does not demand that she shall have personally nursed an example of every case she shall hereafter be called upon to attend. She is not responsible for treatment. She is to be trained to obey orders intelligently. And if she has learned to do this from the point of view both of the surgeon and the physician, is she not a well-trained nurse, even if she has never seen a case of measles nor nursed a gunshot wound?

